# Chimeric Investigations into the Diamide Binding Site on the Lepidopteran Ryanodine Receptor

**DOI:** 10.3390/ijms222313033

**Published:** 2021-12-02

**Authors:** Ewan Richardson, Bartek J. Troczka, Oliver Gutbrod, Ulrich Ebbinghaus-Kintscher, Martin S. Williamson, Christopher H. George, Ralf Nauen, Thomas G. Emyr Davies

**Affiliations:** 1 Department of Biointeractions and Crop Protection, Rothamsted Research, Harpenden AL5 2JQ, UK; eprichardson1@gmail.com (E.R.); B.Troczka@exeter.ac.uk (B.J.T.); martin.williamson@rothamsted.ac.uk (M.S.W.); 2 Department of Biosciences, College of Life and Environmental Sciences, Penryn Campus, University of Exeter, Penryn TR10 9FW, UK; 3 Bayer AG, Crop Science Division, R&D, D-40789 Monheim, Germany; oliver.gutbrod@bayer.com (O.G.); ulrich.ebbinghaus-kintscher@bayer.com (U.E.-K.); ralf.nauen@bayer.com (R.N.); 4 Institute of Life Sciences, Swansea University Medical School, Swansea SA2 8PP, UK; christopher.george@swansea.ac.uk

**Keywords:** diamide insecticides, chlorantraniliprole, flubendiamide, lepidoptera, *Plutella xylostella*, binding site

## Abstract

Alterations to amino acid residues G4946 and I4790, associated with resistance to diamide insecticides, suggests a location of diamide interaction within the pVSD voltage sensor-like domain of the insect ryanodine receptor (RyR). To further delineate the interaction site(s), targeted alterations were made within the same pVSD region on the diamondback moth (*Plutella xylostella*) RyR channel. The editing of five amino acid positions to match those found in the diamide insensitive skeletal RyR1 of humans (hRyR1) in order to generate a human–*Plutella* chimeric construct showed that these alterations strongly reduce diamide efficacy when introduced in combination but cause only minor reductions when introduced individually. It is concluded that the sites of diamide interaction on insect RyRs lie proximal to the voltage sensor-like domain of the RyR and that the main site of interaction is at residues K4700, Y4701, I4790 and S4919 in the S1 to S4 transmembrane domains.

## 1. Introduction

Diamides are a relatively new class of synthetic insecticidal compounds which act on the nerve–muscle boundary, causing contraction and paralysis of insect muscle cells. Members of the class vary in their spectrum of control, but the majority of these insecticides display extremely clean toxicological profiles. Diamide insecticides target the insect’s ryanodine receptor (RyR) and are highly effective against a variety of insect pests (especially those in the order Lepidoptera) but have little effect against mammalian RyRs. Characterisation of the concentration–response relationship in various mammalian (mouse C2C12, rat PC12) cell lines expressing RyRs and recombinant cells expressing insect RyRs indicated chlorantraniliprole (CLR) to be some 300-fold less potent against mouse skeletal muscle RyR (RyR1) and >2000-fold less potent against rat cardiac muscle RyR2 compared to lepidopteran and dipteran RyR-expressing cells [1]. Similarly, flubendiamide (FLB) exhibits >500-fold differential selectivity toward insect over mammalian (rat PC12) receptors [2].

Differences in diamide efficacy between mammals and insects are suspected to be attributable to differences in the structure of the diamide binding site on the RyR. The approximate diamide binding region on the RyR has been elucidated progressively over the past decade, primarily as a by-product of investigations into incidences of field-resistance to diamide insecticides, uncovering a selection of closely located, causatively linked, point mutations on the RyR of lepidopteran pests, centred around residues G4946 and I4790 (*Plutella xylostella* numbering) (reviewed in [3]). Additionally, an earlier study by Tao et al. [4] bears further detailed discussion, as it has been instrumental in pinpointing the diamide binding region. By creating a chimeric RyR channel, composed of the *Drosophila melanogaster* (fruit fly) and *Meloidogyne incognita* (root knot nematode) RyR sequence, a region within the C-terminus of the receptor was found to be critically involved in the formation of the diamide binding site, in line with the findings of a previous study [5]. A defining shorter chimeric segment within this C-terminal region, consisting of a 45aa region of the nematode sequence, located proximal to transmembrane segment S1 (corresponding to *P. xylostella* aa numbering 4659–4703), produced a channel that was insensitive to CLR (up to 30 µM). Of the 45 amino acids replaced, most are unlikely candidates to be involved in diamide interaction. The first 30 aa lie in a zone of high sequence divergence, even within insects, hence the conservation of a diamide binding site here would seem improbable. The last 10 amino acids (aa 4694–4703), by contrast, are highly conserved, likely due to being at the start of the S1 membrane-spanning domain. Six of these amino acids are identical between humans and insects, leaving just K4695N, K4700R, Y4701F and V4702L (*P. xylostella* numbering) as possible loci of this major difference between species in diamide binding efficacy. The latest episodes of diamide resistance reported in the rice stem borer (*Chilo suppressalis*) add further support to the Tao et al. study [4], with alleles 4701C and 4701D found (alone) in the resistant population [6]. The importance of these positions was recently also highlighted by Ma et al. [7] in an analysis of a high-resolution cryo-electron microscopy (cryo-EM) structure of rabbit RyR1 in complex with CLR.

To further delineate the diamide interaction site(s) on the lepidopteran RyR channel, targeted alterations were made to five amino acid positions (including K4700 and Y4701) located within transmembrane-spanning regions S1 to S4 of the voltage sensor-like domain on the *P. xylostella* RyR to match those found in the skeletal RyR1 of humans (hRyR1), generating a human–*Plutella* chimeric construct. This modified RyR construct was then expressed in vitro and challenged with diamide insecticides to assess the degree of sensitivity of the channel to the insecticides.

## 2. Results

**Figure 1:**
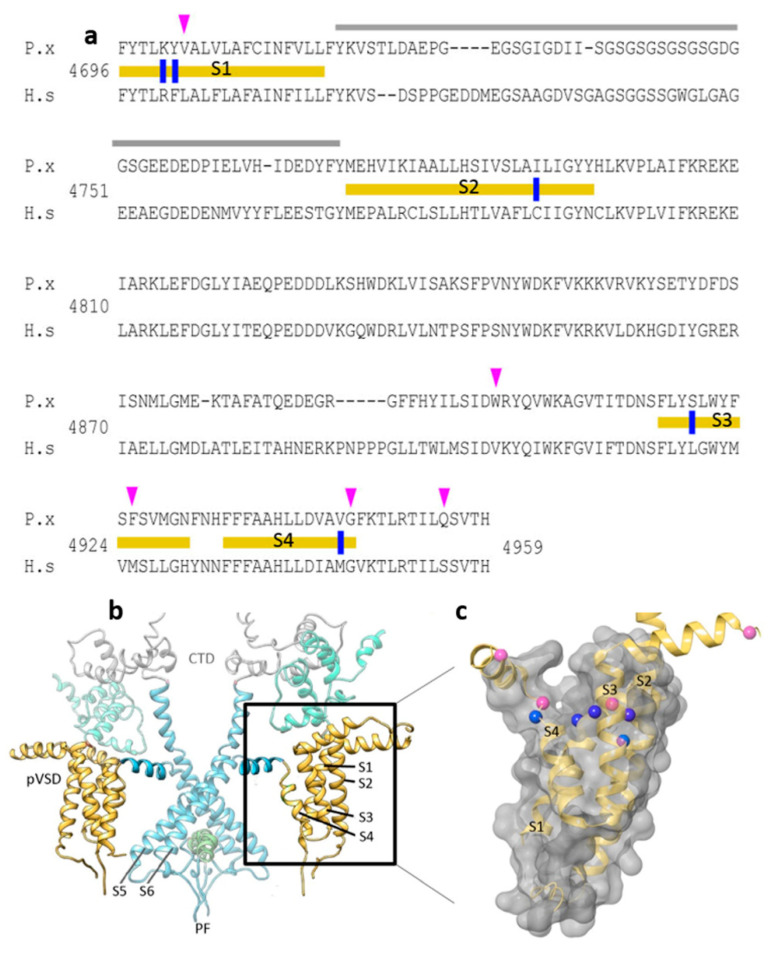
The ‘diamide resistance region’ in lepidopteran RyR. (**a**) Alignment of *Plutella xylostella* PxRyR (P.x) and human skeletal muscle hRyR1 (H.s) amino acid sequences. TM regions S1–S4 (delineated with yellow bars), divergent region (grey), aa residues investigated in this paper (blue), further residues that may be of additional interest based on the amino acid alignments (pink). (**b**) PxRyR-rRyR1 homology model of the RyR transmembrane region showing two isomers in dimeric formation, with the pore region shown in blue (PF, Pore Forming; CTD, C-terminal Domain; pVSD, Voltage Sensor Domain) and TM regions S1–S4 shown in yellow (boxed and highlighted). (**c**) PxRyR-rRyR1 homology model of the pVSD (labeled as in (**a**)), displaying a ring of amino acids implicated in diamide binding (blue) and further residues that may be of additional interest based on the amino acid alignments (pink) but which were not included in the current study. The PxRyR-rRyR1 homology model was generated using Pymol and Schrodinger software.

In the current study, candidate diamide interacting residues on the insect RyR channel identified in previous studies were further scrutinized and assessed through alignment of 44 arthropod, nematode and vertebrate RyR amino acid sequences. Highly conserved but distinct insect vs. mammalian residues K4700R and Y4701F (located on TMS1), I4790C (TMS2), S4919L (TMS3) and V4945M (TMS4) (Figure 1a), were considered suitable candidates for further study. The justifications for their selection are summarized in Table 1.

Notably, almost all the diamide resistance-associated mutations discovered to date (e.g., G4946E/V, I4790M/K, Y4701C/D) are concentrated within a 250 aa region close to the C-terminus of the protein, which in the 3D structure is located within the TM S1–S4 domain, peripheral to the channel pore (Figure 1b,c). This 250 aa region, which is similar in structure to the voltage sensor domain (VSD) of voltage-gated channels such as the bacterial *KcsA* potassium channel, is referred to here as the ‘diamide resistance region’. The candidate amino acid substitutions identified above for further binding site studies, when plotted onto the 3D structure, can be seen to form a ring-like distribution across the crown of this VSD (Figure 1c).

### 2.1. RFCLM: A Chimeric RyR Channel Combining Moth with Mammal

The methodology chosen to investigate the binding region closely follows that of Tao et al. [4], whereby *Spodoptera frugiperda* Sf9 cells are transiently transfected with moth/human RyR chimeras in order to iteratively refine the residues and locus most responsible for diamide insensitivity in the mammalian channel. To this end, four PxRyR recombinant constructs were expressed in Sf9 cells and evaluated in terms of diamide effect relative to WT.

**Figure 2:**
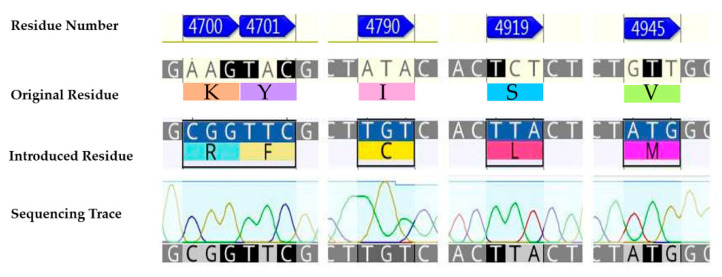
The five aa residue changes investigated: K4700R; Y4701F; I4790C; S4919L; V4945M.

In the first instance, a chimeric construct composed of the wild-type *P. xylostella* (WT-PxRyR) incorporating five amino acid alterations was created, edited to match those residues present in human or rabbit (*Oryctolagus cuniculus*) RyR1. The chimeric construct is hereby referred to as RFCLM-PxRyR, reflecting the five alterations: K4700R; Y4701F; I4790C; S4919L; V4945M (Figure 2).

### 2.2. Characterisation of RFCLM Caffeine Response

**Figure 3:**
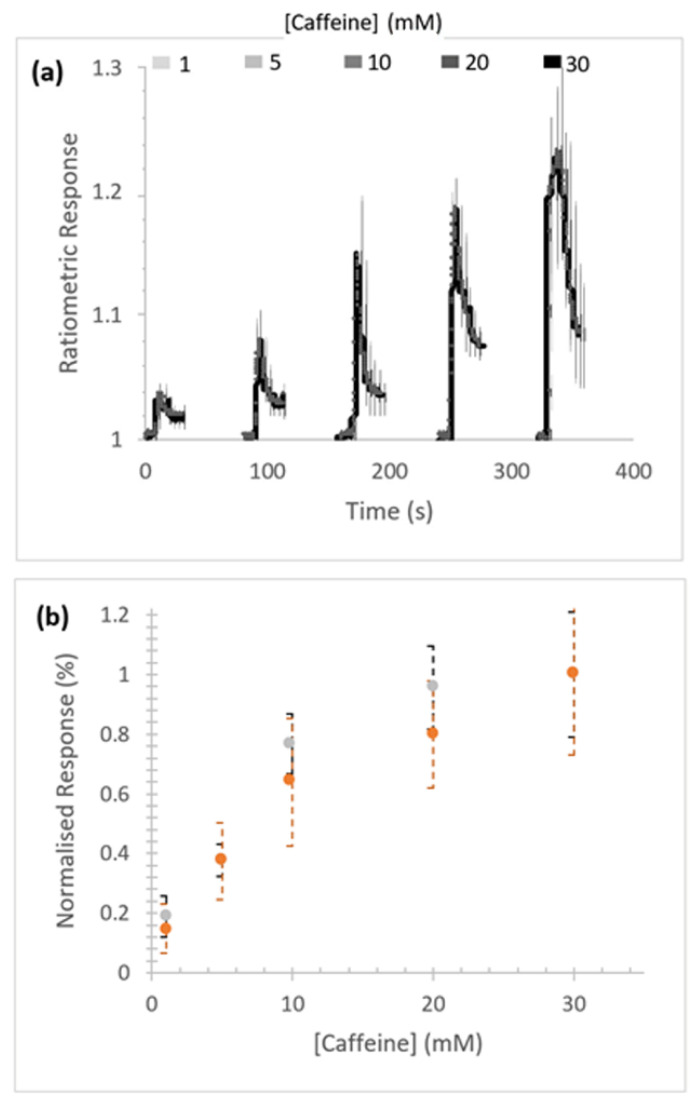
Response of RFCLM-PxRyR to increasing concentrations of caffeine. (**a**) Averaged response of cells in a given field of view. Data is presented as mean (solid line) and standard error of the mean (SEM). (**b**) Proportional normalised responses (see Methods) of those same RFCLM-expressing cells (orange) compared alongside WT-expressing cells (grey). Error bars are standard error of mean (SEM).

Concentration-dependent caffeine-stimulated Ca^2+^ release was measured in Sf9 cells expressing homotetrameric RFCLM-PxRyR (Figure 3a). No substantial difference in signal amplitude between the WT and RFCLM expressed constructs in response to caffeine was indicated within the tested range (Figure 3b).

### 2.3. Characterisation of RFCLM Diamide-Response

**Figure 4:**
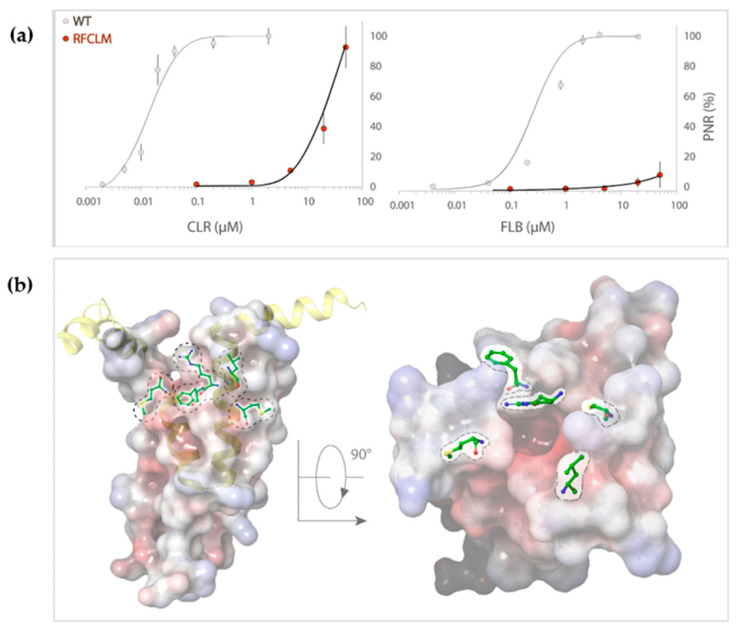
(**a**) RFCLM-PxRyR is only activated by CLR at very high concentrations (>10 µM) but is not activated by FLB at its limit of solubility. Graphs display the dose–response relationship of RFCLM-PxRyR (red fill) to CLR and FLB (with WT PxRyR (grey fill) response for comparison). CLR- and FLB-induced Ca^2+^ release was normalised to the maximal caffeine-evoked Ca^2+^ release in the same cells to calculate the proportional normalised response (PNR), which is presented here as % maximum PNR. (**b**) PxRyR-rRyR1 homology model of the RFCLM-PxRyR pVSD, displayed in (i) longitudinal and (ii) transverse orientation. Positions of the five amino acid substitutions are marked; dotted lines indicate that the residue is hidden within the structure. The homology model was generated using Pymol and Schrodinger software.

Concentration-response experiments were conducted for RFCLM-PxRyR against CLR and FLB. Poor solubility limited the maximum applied concentration for both diamides to 50 µM. A WT-PxRyR EC_50_ of 0.015 µM for CLR and 0.27 µM for FLB are comparable to those generated by previous authors [8] (EC_50_s = 0.017 µM for CLR; 0.25 µM for FLB). RFCLM-PxRyR demonstrated a decreased sensitivity to CLR compared to the WT-PxRyR construct and near elimination of FLB-responsiveness (Figure 4a). An approximate minimum ‘sensitivity ratio’ (SR) for CLR was ascertained by comparison of WT-PxRyR EC_50_ (0.015 µM) with the ‘minimum EC_50_’ of RFCLM-PxRyR (20.4 µM), giving a SR = 1360-fold. In the case of FLB (sulfoxide form), the responses were small and transient up to and beyond the limit of solubility of the compound (Figure 4a). RFCLM-PxRyR expressing cells did at no point display a typical, irreversible activation response to FLB. The closest approximation to an SR, made by comparing the EC_10_ for WT (50nM) with that for RFCLM (50 µM), gives an approximate SR of 1000-fold for FLB.

The impact of these five amino acid changes upon diamide efficacy is shown to be extreme where diamide interaction is all but abolished, creating a channel similar in diamide interaction properties to the human hRyR channel itself (RFCLM EC_50_ for CLR = 20.4 µM, which is comparable to studies in mice [1]). The results confirm the location of the diamide interaction site as being within the voltage sensor-like domain. Figure 4b indicates the relative positions of the five modified residues in the pVSD region, where they encircle a cavity of high electronegativity. Such a region of high electronegativity, surrounded by an opening of neutral and electropositive residues, is a common feature of voltage-gated ion channels, frequently representing the site of ligand interaction.

### 2.4. Unpicking the RFCLM Modification

In pursuit of identifying which of the five amino acid residues in WT-PxRyR contributes most significantly to the lepidopteran RyR susceptibility to diamides, three of the five RFCLM amino acid substitutions were substituted individually, or in pairs, into the WT-PxRyR construct, and their sensitivity to CLR assessed.

**Figure 5:**
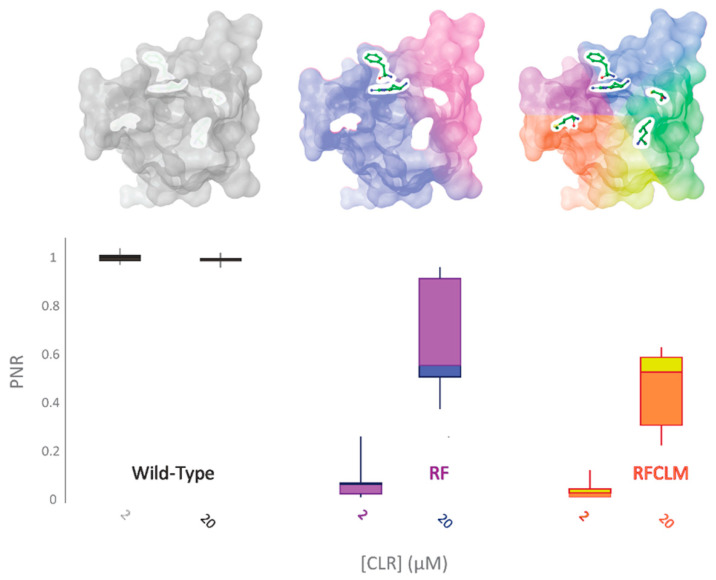
RF-PxRyR and RFCLM-PxRyR share a similar phenotypic response to CLR exposure. Sf9 cells (n = 10) expressing either Wild-Type (grey), RF (blue/purple) or RFCLM (yellow/orange) PxRyR were exposed to 2 µM and 20 µM concentrations of CLR. All diamide responses are expressed as proportional normalised responses (PNRs). Error bars represent SEM.

Of the five altered aa residues, a K4700R-Y4701F (RF-PxRyR) combination was considered a strong candidate to be mediating the observed effects, based on the previous work by Tao et al. [4]. RF-PxRyR, when expressed in Sf9 cells, shows a highly significant reduction in CLR response relative to WT-PxRyR (Figure 5) and suggests that the two amino acid alterations K4700R and Y4701F in tandem are responsible for mediating a majority of the RFCLM-PxRyR phenotype. 

**Figure 6:**
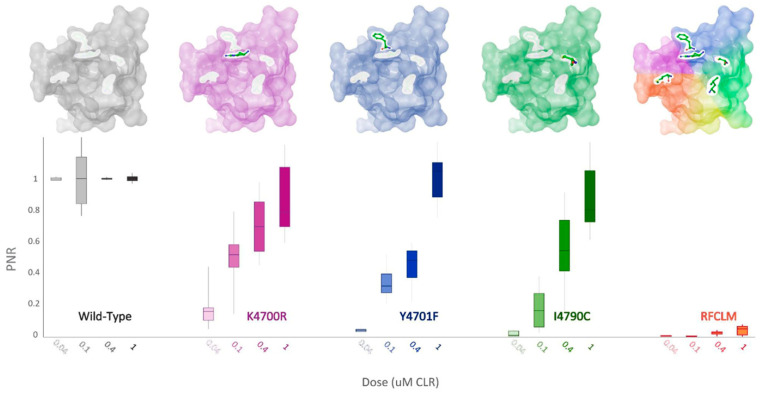
PxRyR variants exhibit differing responses to increasing concentrations of CLR. Sf9 cells (n = 5–26) expressing either Wild-Type (grey), K4700R (purple), Y4701F (blue), I4790C (green) or RFCLM (orange) -PxRyR were exposed to increasing concentrations of CLR. All diamide responses are expressed as proportional normalised responses (PNRs). Error bars represent SEM.

When K4700R, Y4701F and I4790C substitutions were substituted individually into the WT-PxRyR background, all three novel variants were found to confer a significant reduction in diamide response amplitude at the four discriminating doses of CLR tested (Table 2, Figure 6). However, the response magnitude of each individual change was 50–100 times lower than the synergistic effect of all five changes combined (RFCLM-PxRyR). The minimum concentration at which any of the modified PxRyR’s registered a response was 0.04 µM, a dose that exceeds the EC_50_ of the WT-PxRyR (0.015 µM CLR). Y4701F and I4790C do not respond at this concentration and their response is not significantly different from that of RFCLM-PxRyR. The magnitude of the K4700R response at 0.04 µM is 10–20% compared to the WT response, whereas the introduction of either Y4701F or I4790C is associated with a much more robust and significant reduction in CLR efficacy. It is notable that despite K4700R and Y4701F individually giving just a moderate (up to 26-fold) reduction in diamide efficacy, when the two substitutions are combined the resulting RF-PxRyR displays the same diamide-resistant phenotype as RFCLM-PxRyR (RR > 1000-fold).

### 2.5. Assessing the Contribution of Y4701F on Flubendiamide Efficacy

Y4701 alterations (Y4701C/D) have been found in diamide-resistant populations of *C. suppressalis* and are apparently increasing in frequency [6]. Bioassays of these populations indicate that changes at this residue are involved in a 250-fold resistance to CLR. This amino acid change was therefore hypothesised to play a central role in the RF phenotype (Figure 5).

**Figure 7:**
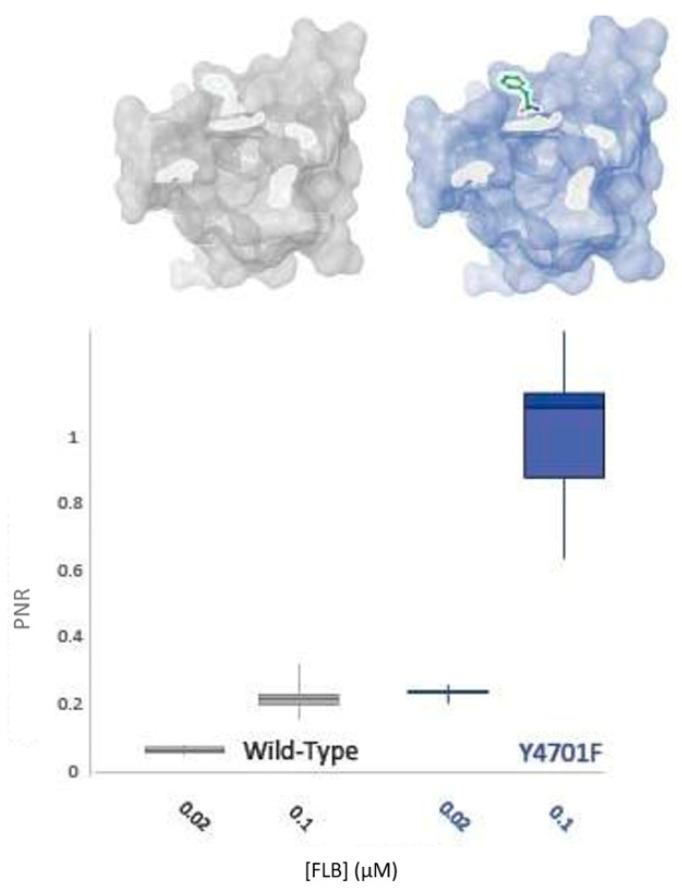
WT-PxRyR and Y4701F-PxRyR exhibit differing responses to low concentrations of FLB. All diamide responses are expressed as proportional normalised responses (PNRs). Error bars represent SEM.

It has been established in this study that this residue appears to play a more significant role in CLR insensitivity than its neighbour, K4700 (Figure 6). However, field studies have indicated that substitutions at Y4701 might have differential effects vs. CLR and FLB [11]. Accordingly, the impact of the residue upon FLB efficacy was tested. As displayed in Figure 7, the Y4701F substitution surprisingly does not confer decreased sensitivity to FLB. Indeed, the alteration may confer an increase in FLB susceptibility by up to 5-fold.

## 3. Discussion

This study builds upon the methodological approach of Tao et al. [4], using a targeted amino acid substitution approach to generate a (*Plutella*–human) RyR chimera to extend knowledge of the diamide binding region on the insect RyR. Using this approach, we identified five amino acid substitutions within human RyR1, referred to as RFCLM (K4700R; Y4701F; I4790C; S4919L; V4945M (*P. xylostella* numbering)), which have a profound impact upon diamide efficacy when introduced in concert into an otherwise WT-PxRyR background. These five alterations when mapped on to a 3D homology model of the RyR protein were shown to be located in close proximity to one another, as well as to well-characterised diamide resistance-associated mutations, and all lie within the previously defined ‘diamide-resistance region’ on the voltage senor-like (pVSD) domain in the transmembrane region of the channel. Further investigations indicated that two of the five residues, K4700R and Y4701F, were together responsible for a reduction in diamide sensitivity of approximately equal magnitude to that generated by the five residues RFCLM in concert. It seems clear from these results that the relatively moderate phenotypes associated with each individual change combine additively, or synergistically, to provide a high level of insensitivity to diamide insecticides.

**Figure 8:**
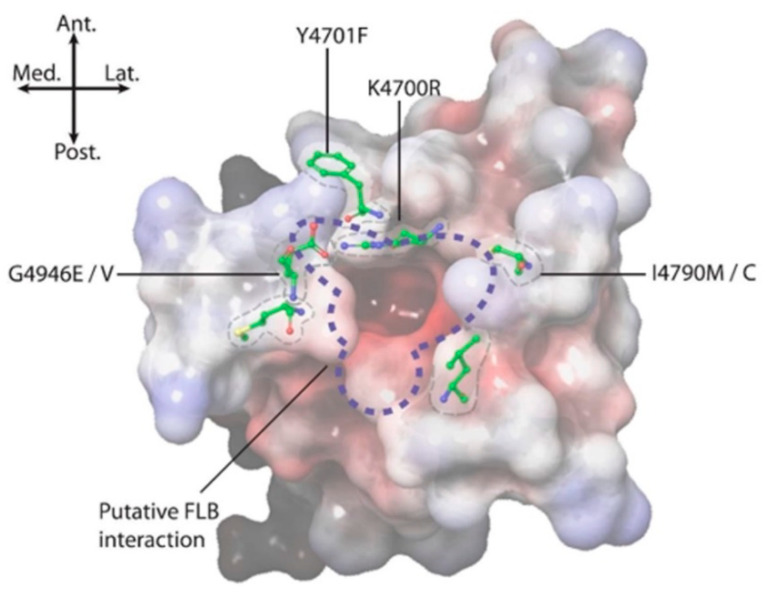
Positional summary of key amino acid residues implicated in diamide binding and insecticide resistance. Electrostatic potential: positive (blue), neutral (white) and negative (red).

Field-derived, resistance-causing mutations, such as G4946E/V [9,12], I4790M/K [10,13], Y4701C/D [6] and Y4922F [11], seem most likely to mediate their effects through topographical alterations to the pVSD environment on the RyR in the immediate vicinity of the diamide binding site (Figure 8). Studying such changes provided useful information in terms of identifying the extent/perimeters of the binding area [3,14]. However, within that binding area, it is important to identify the residues responsible for forming intermolecular forces (IMFs) with the diamide ligand.

A recent study by Ma et al. [7] used a 3D homology model of *P. xylostella* RyR, based on the high-resolution cryo-EM structure of rabbit rRyR1 in the open state in complex with CLR, to identify key points of diamide interaction. This study determined that the CLR molecule when bound induces a conformational change resulting in a displacement of the S4–S5 linker, thereby triggering channel opening. The binding site for CLR, predicted to be located within the pVSD of RyR and facing the cytosol, was further corroborated by mutagenesis data, which revealed how the diamide insecticides are selective to *P. xylostella*. Whereas the pyridine and pyrazole moieties of CLR are responsible for stabilizing the ligand in the pocket, the anthraniloyl moiety accounts for the species-specific binding. The defined binding pocket on rRyR1 has oppositely charged amino acid residues on each end, contributed by R4563 and D4815, respectively (equivalent to residues K4700 and D4942 in *P. xylostella*), which account for the majority (approx. 80%) of the binding energy interaction with diamide insecticides. The modelling data also revealed that several pests have developed resistance to diamide insecticides via two mechanisms, steric hindrance (involving I4790M/K and G4946E/V) and loss of contact (involving Y4701C/D and Y4922F). 

A key residue identified by Ma et al. in their study [7] as being involved in diamide binding on rRyR1 is the positively charged R4563, equivalent to residue K4700 in *P. xylostella*, which was also highlighted in the present study as being a key CLR binding determinant. This insect-specific lysine at position 4700 is most likely largely responsible for the affinity of CLR for *P. xylostella* RyR being ~200-fold higher compared to mammalian RyR1 [15], possibly because it is a less bulky residue that better accommodates CLR in the binding pocket. The *P. xylostella* RyR model also shows that residues Y4701, I4790 and S4919 (further highlighted in our study), as opposed to the equivalent residues F4564, C4657 and L4792 in rRyR1, facilitate increased contact with CLR and results in a more favourable interaction of the RyR binding pocket with the insecticide. This would account for the decreased sensitivity to CLR observed in our experiments when some of these residues were individually substituted for the rRyR1 equivalents in the *P. xylostella* RyR. Three of these residues, K4700, Y4701 and S4919, are completely conserved in all insect species, whereas I4790 is conserved only in the Lepidoptera, the equivalent residue in other insects being a methionine (M), and must therefore confer some degree of specificity. Interestingly, our experiments showed that the substitution Y4701F does not adversely affect binding of FLB but actually enhances the channel’s sensitivity to this compound, suggesting that although the overall binding mode of CLR and FLB are similar, subtle differences in how they interact with the binding pocket clearly exist.

The results presented highlight the toxicological relevance of a selection of amino acid residues around the diamide binding site on *P. xylostella* RyR and extend further the data presented by Ma et al. [7]. At least four of the five residues selected for our study, K4700, Y4701, I4790, S4919, are involved in insect-specific binding of CLR [7]. The fifth residue of the RFCLM quintet, involving the substitution V4945M, although not tested as an individual RyR substitution in our study, is deemed unlikely to be substantially involved in CLR binding, as it is located at the periphery of the binding pocket and faces away from the binding site, as defined in the 3D RyR model.

## 4. Materials and Methods

### 4.1. Chemicals

Chemicals used for the preparation of bacterial media were purchased from Sigma (Sigma-Aldrich, St. Louis, MO, USA). Analytical grade dimethyl sulfoxide (DMSO, purity ≥ 99%) used for dilution of all active compounds was obtained from Sigma. Technical grade flubendiamide sulfoxide and chlorantraniliprole (purity > 98%) was provided in-house (Bayer CropScience, Monheim am Rhein, Germany) or purchased as analytical standard from Fluka Chemicals (Buchs, Switzerland), respectively. Analytical grade caffeine was purchased from ReagentPlus® (Sigma).

### 4.2. Mutagenesis of PxRyR

Construction of the pIZ-WT-PxRyR/V5-His plasmid used in this study is as described in Troczka et al. [8]. The novel PxRyR modifications introduced during this study are listed in Supplementary Table S1, along with the oligonucleotide sequences used to introduce the change. Prior to mutagenesis, the 1,8177 bp pIZ-WT-PxRyR/V5-His construct was digested into five fragments (detailed in Supplementary Figure S1) and each fragment was separately incorporated into a pcDNA3.1(-) vector. All the listed changes in Supplementary Table S1 are to the C-fragment. A modification was made to the protocol previously described in [8] to facilitate ease of fragment re-assembly (details available in Supplementary Methods M1). Transformed, purified PxRyR assemblies were validated for completeness via diagnostic digestion, complete amplification and complete Sanger sequencing (Eurofins Genomics, Wolverhampton, UK). The RFCLM-PxRyR modification (containing the five alterations: K4700R; Y4701F; I4790C; S4919L; V4945M) was generated using a Quick-Change Lightning Multi kit (Agilent, Santa Clara, CA, USA), which allows the introduction of up to five mutations simultaneously into an <8 kb plasmid. The protocol for the Lightning Multi kit differs from the standard protocol above only in primer design, whereby two or more (up to five) primers are used, with each primer capable of introducing one or more changes. Uniquely, all the primers bind to one cDNA strand, with no complementary primer binding required; instead, the *Pfu* enzyme extends the sequence from each primer, in non-overlapping fashion, before knitting together the fragments to generate a single-stranded DNA plasmid for transformation. 

### 4.3. Sf9 Transfection Protocol

*Spodoptera frugiperda* Sf9 cells (Life Technologies, Carlsbad, CA, USA) were grown at 27 °C in Sf900^TM^ II serum-free medium (SFM) (Gibco-Thermo Fisher Scientific, Waltham, MA, USA) in 30 mL suspension cultures supplemented with 0.6% FBS (Gibco-Thermo Fisher Scientific). Transfection of cells with the pIZ-PxRyR/V5-His expression plasmid and Cellfectin^TM^ (Thermo Fisher Scientific) was performed according to the manufacturer’s (Thermo Fisher Scientific) instructions. Glass coverslips (1 cm^2^ diameter) coated with Poly-L-lysine (Sigma, Burlington, MA, USA) were placed in a 4-well plate. Each well was then filled with 0.5 mL of Sf-900^TM^ II medium and each coverslip was seeded with 150,000 cells, at a density of 800 cells/mm^2^ to produce an approximately 90% confluent monolayer. Cells were allowed to attach to the coverslips for 16 h and then were transfected. Transfection solution was composed of 3.25 µg pIZ-PxRyR/V5-His plasmid DNA dissolved in water; 4.5 μL PLUS^TM^ enhancer reagent (Thermo Fisher Scientific); 20 μL Cellfectin^TM^ (Thermo Fisher Scientific); per 1 mL of fresh Sf900^TM^ II SFM. The Cellfectin and DNA:PLUS solutions were individually mixed and incubated for 5 min, before being combined and incubated for a further 30 min. Cells were removed from their media and washed twice, prior to the addition of transfection solution. Transfection incubations proceeded for 4 h before the cells were washed and returned to 30% conditioned SF900^TM^ II SFM, with 0.6% FBS. Post-transfection, cells were incubated at 27 °C for 40–52 h. 

### 4.4. Calcium Imaging and Data Collection

Fura 2-AM dye (Life Technologies, Carlsbad, CA, USA) was used for monitoring calcium release in Sf9 cells transfected with recombinant PxRyR. Cells were loaded with Fura 2-AM calcium sensitive dye 48 h post transfection. Cells on coverslips in 4-well plates were first put into 500 µL of fresh SF-900^TM^ II SFM and then 2 µL of the dye stock solution (1 mM) was added (to generate a final concentration of 4 µM). Cells were left to incubate at 27 °C for 45–60 min, followed by 3 washes with 500 µL of fresh unsupplemented SF-900^TM^ II SFM. Prior to imaging, coverslips with Fura-2-AM loaded cells were placed in standard Ringer’s solution, with 2 mM [Ca^2+^] (CaCl_2_). All experiments were carried out in an air-conditioned room maintained at approximately 25 °C. Data collection for all the calcium imaging studies took place on a Ratiometric Imaging Perfusion system (RIPS), utilizing an Axio Vert.A1 microscope with a LD Plan-Neofluar ×10/0.4 lens (Zeiss, Oberkochen, Germany), measuring the ratio of excitation at 340/380 nm (calcium free/calcium bound indicator) every 180 ms and capturing emission at 510 nm. Cells on the coverslip were placed into a perfusion chamber of approximately 0.5 mL volume mounted on the microscope stage. Continuous unidirectional flow of Ringer’s through the bath was driven by a peristaltic pump, allowing for a constant fluid exchange. Caffeine and diamide agonist test solutions were applied in 3 s bursts using a metal U-tube. Fluid dynamics were measured using a solution of red amaranth dye, diluted 1:20 in Ringer’s. Perfusion flow rate was 49 µL/s. Experiments on cells consisted of multiple agonist applications, with the order and timing of applications dependent on the experimental aims. Recordings began at T = 0 s, with application of 10 mM caffeine at 7–10 s, followed by a 140 s delay, during which period caffeine-responsive cells in the field-of-view (FOV) were identified (i.e., those cells exhibiting a large biphasic Ca^2+^ event following the application of caffeine). During concentration–response experiments a single caffeine application was followed by a single diamide application at 150 s. Measurements were taken for all four PxRyR constructs in a single day in order to minimise methodological variation, with between 6–25 cells responding for each construct. Experiments were recorded using VisiView® (Visitron Systems, Puchheim, Germany) software. Raw video capture on the software was used to identify caffeine-responsive cells and assess response mode. Outputted numerical pixel intensity data were analysed using Microsoft Excel and SigmaPlot v.12 (Systat Software, Chicago, IL, USA).

### 4.5. Agonist Diluent and Background Fluorescence

Due to their low solubility in water, stock PxRyR agonists used in this study were dissolved initially in DMSO and then further diluted into Ringer’s medium at a dilution factor of 1:100. As the 1% DMSO was found to cause a gradual rise in baseline Ca^2+^ in some Sf9 cells (both transfected and non-transfected), the fluorescence amplitude of cells exhibiting no change in fluorescence in response to caffeine was measured during each diamide measurement and then subtracted from the fluorescence of each responding cell in order to adjust for any DMSO-mediated fluorescence. Similarly, changes in background fluorescence due to application of agonist frequently occur in non-ratiometric calcium imaging, either due to changes in solution viscosity or due to poor dispersal of agonist in the media. In this present case, use of a ratiometric dye compensated for the issue of background disturbance, and the effects occur equally at both ratiometric wavelengths and therefore cancel each other out. Additionally, pluronic F68 (Gibco-Thermo Fischer Scientific) was added to the final solutions of all agonists (including caffeine) at 0.003% concentration, in order to aid the solubility of the diamide compounds [16]. 

### 4.6. Calculating Proportional Normalised Response (PNR) of Individual Cells to Diamide Insecticides 

Responses of individual cells to the application of caffeine and then diamide compounds were recorded. Diamide response amplitude was normalised to the prior caffeine response to create a response ratio, which was then normalised against the maximal caffeine responses to establish the proportional normalised response (PNR). In brief, to calculate the PNR, raw data was normalized using the equation: R/R_0_, where R is the fluorescence ratio value recorded for an individual cell upon each individual time point and R_0_ is an average fluorescence ratio calculated over the first 5 s prior to addition of the agonist. The maximum response amplitude is taken as the maximum fluorescence signal outputted by the cell across all time frames. Final amplitude data is presented as a mean value and the standard deviation of the mean. In all the concentration–response plots, response data is expressed as a percentage of the highest response registered. The magnitude of Ca^2+^ release occurring in response to diamide addition was normalized to the initial caffeine-evoked Ca^2+^ release in the same cell (10 mM caffeine application occurs 150 s before diamide application). A full description of the data analysis pipeline employed is provided in Appendix A.

## Figures and Tables

**Table 1:** PxRyR amino acid positions altered to resemble hRyR1. The actual residue at each position is noted for susceptible (insect, arthropod) and non-susceptible (vertebrate, nematode) species.

Position of Modification	Residue in Susceptible Insect or Arthropod Species	Residue in Resistant Species	Additional Justification for Selection of Residue
4945	Valine (V)	Methionine (M) in vertebrates Leucine (L) in nematodes	Sequentially adjacent to G4946E [8,9]
4919	Serine (S) or asparagine (N)	Leucine (L) in vertebrates Arginine (R) in nematodes	
4790	Isoleucine (I)	Leucine (L) in vertebrates Cysteine (C) in nematodes	Methionine (M) at this position shown to confer diamide resistance [10]
4701	Tyrosine (Y)	Phenylalanine (F) or methionine (M) in vertebrates Lysine (K) in nematodes	Terminal residue of the Tao chimera [4]
4700	Lysine (K)	Arginine (R) in vertebrates Glutamate (E) in nematodes	Penultimate residue of the Tao chimera [4]

**Table 2:** Comparison of the responses of PxRyR sequence variants to discriminating CLR concentrations (**a**–**d**). Grey boxes list the average response of that construct (PNR). White boxes display indices of significance, based on LSD comparisons of amplitude between the variants. N.S. = Not Significant; * = *p* < 0.05; *** = *p* < 0.001. The least significant difference (LSD) consists of a pairwise comparison of mean average response amplitude between the constructs compared to the standard deviation of all groups combined.

(a) 0.04 µM CLR						(b) 0.1 µM CLR					
	WT	K4700R	Y4701F	I4790C	RFCLM		WT	K4700R	Y4701F	I4790C	RFCLM
WT	1	***	***	***	***	WT	1	***	***	***	***
K4700R		0.2	***	***	***	K4700R		0.51	*	***	***
Y4701F			0.039	N.S.	N.S.	Y4701F			0.33	*	***
I4790C				0.021	N.S.	I4790C				0.18	*
RFCLM					0.0061	RFCLM					0.0038
**(c) 0.4 µM CLR**						**(d) 1 µM CLR**					
	**WT**	**K4700R**	**Y4701F**	**I4790C**	**RFCLM**		**WT**	**K4700R**	**Y4701F**	**I4790C**	**RFCLM**
WT	1	*	***	***	***	WT	0.99	N.S.	N.S.	N.S.	***
K4700R		0.69	*	N.S.	***	K4700R		0.96	N.S.	N.S.	***
Y4701F			0.43	N.S.	***	Y4701F			1.039	N.S.	***
I4790C				0.55	***	I4790C				0.89	***
RFCLM					0.025	RFCLM					0.042

## Data Availability

Data is contained within the article or Supplementary Materials.

## Supplementary Materials

The following are available online at https://www.mdpi.com/article/10.3390/ijms222313033/s1.

## Appendix A

A data analysis pipeline consisting of the following steps was used to calculate the PNR to diamide insecticides for each responding cell.

- Identify caffeine-responsive cells;

For each cell, the caffeine response was measured as follows:

▪ Calculate ‘average fluorescence prior to application’: R_0_
▪ Calculate ‘normalised fluorescence score at each timepoint’ for responding cells: R/R_0_^response^
▪ Calculate and subtract ‘cellular background’: (R/R_0_) ^total^ = (R/R_0_) ^response^ − (R/R_0_)^non-response^
▪ Calculate ‘maximum fluorescence amplitude’: ∆(R/R_0_) (Caffeine) = Max(R/R_0_)^total^ − 1.

Similarly, measure response to diamide as follows:

▪ Calculate R_0_;
▪ Calculate R/R_0_;
▪ Calculate ∆R/R_0_ (Diamide);
▪ For each cell, divide ∆R/R_0_ (Diamide/Caffeine) to get the PNR triggered by diamide.

The above was repeated for all cells in the FOV for the given dose, and the mean average PNR across all cells in the FOV was calculated.
