# Supplementary material for: Chimeric Investigations into the Diamide Binding Site on the Lepidopteran Ryanodine Receptor
Source: Int J Mol Sci. 2021 Dec 2;22(23):13033. doi: 10.3390/ijms222313033 (PMC8657592; doi:10.3390/ijms222313033)
Supplement: Supplementary file 1 [file ijms-22-13033-s001.zip › ijms-1482705-supplementary.pdf]

# Supplementary Data

### *Supplementary Methods M1*

To facilitate fragment reassembly, a hybrid plasmid vector was used, composed of the multiple-cloning-site (MCS) from pcDNA3.1(-) (Invitrogen, Carlsbad, CA, USA), plus the addition of two extra restriction enzyme cut sites (HindIII and MluI cut sites) at the 5′ and 3′ ends of the MCS respectively, spliced into a pIZ/V5-His plasmid vector (Thermo-Fisher Scientific, Waltham, MA, USA) in place of its original MCS. The four fragments (N, M1, M2 and a modified C) to be re-incorporated into the linearised hybrid pIZ/V5-His plasmid vector where then re-assembled into a whole intact full-length cDNA corresponding to the PxRyR ORF by ligation in the presence of the cut hybrid pIZ/V5-His plasmid vector. The total DNA concentration in the ligation reaction was maintained at ~10ng/ul. The addition of HindIII and MluI cut sites at the 5′ and 3′ ends of the pcDNA3.1(-) MCS, allowed the amplified sequence reassembled in the hybrid vector to be easily transferred across into pIZ/V5-His.

**Supplementary Table S1:** Primers used to generate amino acid substitutions in the *P. xylostella* RyR.

| Mutation | Oligonucleotide 1 | Oligonucleotide 2 |
|---|---|---|
| **K4700R** | GTTCTACACCTTGcgGTACGTGGCGCTGG | CCAGCGCCACGTACcgCAAGGTGTAGAAC |
| **Y4701F** | GTTCTACACCTTGAAGTtCGTGGCGCTGG | CCAGCGCCACGaACTTCAAGGTGTAGAAC |
| **I4790C** | GTATCGCTGGCTtgtCTGATCGGGTACTAGGATTTGAAGG | CCTTCAAATGGTAGTACCCGATCAGacaAGCCAGCGATAC |
| **S4919L** | CTCTTTCCTGTACTtaCTGTGGTACTTCTCGTTCTCTGTGATGGGC | GCCCATCACAGAGAACGAGAAGTACCACAGtaAGTACAGGAAAGAG |
| **V4945M** | CGCTCATCTGTTGGACGTGGCTaTgGGGTTCAAGACGTTGAGG | CCTCAACGTCTTGAACCCcAtAGCCACGTCCAACAGATGAGCG |
| **RFCLM (MULTI)** | CCTCGCCAGGAAGTTCTACACCTTGAAGTACGTGGCGCTGGTGCTGGCC<br>GCACTCTATAGTATCGCTGGCTATACTGATCGGGTACTACCATTTGAAGG TCCCGC<br>CGATCACAGACAACTCTTTCCTGTACTCTCTGTGGTACTTCTCGTTCTCTGT | No reverse primer required in Lightning Multi Reaction |

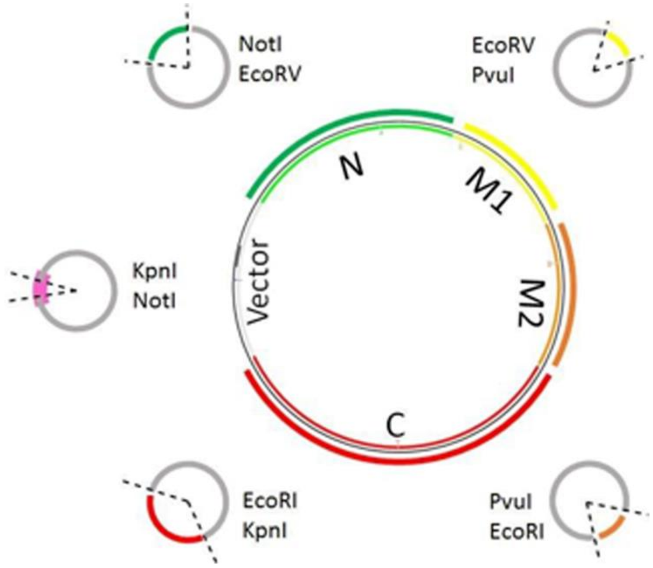


**Supplementary Figure S1:** *PxRyR* construct fragment assembly and restriction sites used for cutting out and re-ligating. The WT-PxRyR sequence was digested into four fragments, as labelled in the diagram. N ('N-terminus', green) - 4061bp M1 (yellow) - 2350bp M2 (orange) - 2684bp C ('C-terminus', red) - 6326bp Vector (pIZ/V5-His) - 2900bp
